# History and epidemiology of foot-and-mouth disease in Afghanistan: a retrospective study

**DOI:** 10.1186/s12917-019-2119-y

**Published:** 2019-10-15

**Authors:** Arash Osmani, Ian Duncan Robertson, Ihab Habib, Ahmad Arash Aslami

**Affiliations:** 1 School of Veterinary Medicine, College of Science, Health, Engineering and Education, Murdoch University, Perth, 6150 Australia; 2 China-Australia Joint Research and Training Center for Veterinary Epidemiology, Huazhong Agricultural University, Wuhan, China; 3 Veterinary Medicine Department, College of Food and Agriculture, United Arab Emirates University (UAEU), P.O. Box 15551, Al Ain, Abu Dhabi, UAE; 4 Central Veterinary Diagnostic and Research Laboratory, Darulaman, Kabul, Afghanistan; 5 Project of Controlling Transboundary Animal Diseases, UN-FAO, Darulaman, Kabul, Afghanistan

**Keywords:** Afghanistan, Epidemiology, Foot and mouth disease, Passive surveillance, Retrospective study

## Abstract

**Background:**

Foot and mouth disease (FMD) is endemic in Afghanistan with serotypes O, A and Asia 1 being prevalent. A retrospective study of data collected through passive surveillance of outbreaks of FMD in Afghanistan from 1995 to 2016 was undertaken to determine the temporal and spatial distribution of FMD in the country.

**Results:**

A total of 4171 outbreaks were reported between 1995 and 2008 with a strong correlation between the number of outbreaks and the number of provinces (*r* = 0.85, s = 68.2, *p* < 0.001); and between the number of outbreaks and the number of districts containing infected animals (*r* = 0.68, s = 147.8, *p* = 0.008). Of 7558 samples collected from livestock originating from 34 provinces in 2009, 2011 and 2013–2015, 54.1% were test positive (FMDV 3ABC-trapping ELISA) and the prevalence varied significantly between years (χ^2^ = 263.98, df = 4, *P* < 0.001). Clinically suspected cases were reported in 2016 with a substantial positive correlation (*r* = 0.70, *P* < 0.001) between the number of districts with cases and the number of reported cases. Serotype O was the predominant serotype detected during the study period, although serotypes A and Asia1 were also detected. Cattle were involved in all outbreaks in the study period and infections were detected in all years of the study in Hirat province in the north-west (bordering Iran), Nangarhar province in the east (bordering Pakistan) and Kabul province in the centre of the country.

**Conclusions:**

The current paper was the first analysis of existing data focusing on the spatiotemporal distribution of FMD in Afghanistan. The findings from this study provide valuable direction for further research to understand the epidemiology of FMD and its control in Afghanistan.

## Background

Foot and mouth disease is endemic in Afghanistan [1–4] with infection predominantly caused by serotype O, although serotypes A and Asia-1 have also been reported [1, 2, 5, 6]. Unpublished reports from the Directorate of Veterinary Services of the Ministry of Agriculture, Irrigation and Livestock (MAIL) indicate that FMD was first recorded in Afghanistan in 1962. However, earlier accounts of FMD virus type A in 1956 in the country have been made [7] and the disease was first reported in neighboring Pakistan in 1954 [4], and consequently it is likely that disease was present in the country prior to these reports.

Agriculture and, in particular, livestock are key components of Afghanistan’s economy [8]. The raising of livestock is common throughout Afghanistan with the major livestock products being wool, hides, fat, mutton, milk and milk products (cream, butter, curd, dry curd, yogurt, ghee and cheese) [8, 9]. Smallholder farming households constitute over 80% of Afghanistan’s population and include 90% of its poor population [10].

According to the FAO [11] there were 3.7 million cattle, 8.8 million sheep, 7.3 million goats, 1.6 million donkeys, 0.2 million camels, 0.1 million horses and over 13 million poultry in Afghanistan when the first and only livestock census was conducted in 2003. Cattle are reared by almost all farmers with even the smallest and poorest farmers keeping at least one dairy cow [8, 12].

The major impact of FMD in Afghanistan, as with most other infected countries, is not only associated with loss of trading opportunities for livestock and their products, but also the direct effects of losses arising from decreased milk yield, abortions, death of young animals and loss of draught power [13]. Factors, including technical issues with handling vaccines, inaccurate diagnostic tests, a lack of political commitment to provide animal health infrastructure, and long porous borders with Pakistan, Iran and Tajikistan, all contribute to the endemic status of FMD in the country. Additionally, due to the limited activities conducted on FMD by Government and Non-Governmental Agencies (NGOs), primarily because of the long-standing conflicts making many locations unsafe to visit, along with their geographical remoteness, has hindered the delivery of effective veterinary services to the livestock sector. Therefore, it is likely that historical disease records are incomplete resulting in underreporting of disease. Others [2, 14] have similarly noted a lack of reliable data on FMD and its distribution in Afghanistan, primarily due to an inefficient surveillance system.

Currently the diagnostic test used for FMD in Afghanistan is a trapping-indirect ELISA for the detection of antibodies to the non-structural (NS) polypeptide 3ABC of FMDV in serum or plasma samples from large and small ruminants. Until 2004 all samples collected were sent to FMD reference laboratories (Padova in Rome Italy and Pirbright Institute in the UK) for diagnosis. Even though the Central Veterinary Diagnostic and Research Laboratory (CVDRL) had the ability to run diagnostic tests from 2004 onwards, serum and tissue samples were still sent to the two international reference laboratories for confirmation and genotyping of the FMDV until 2011. From 2011 onwards the CVDRL has been able to detect and genotype FMDV. However, due to the unavailability of reagents in the country and the high price of the ELISA test kits, the government is unable to purchase sufficient diagnostic kits and consequently relies upon the financial aid of international organizations, such as the Food and Agricultural Organization of the United Nations (UNFAO), to support the diagnosis of the disease in its livestock.

For the successful control of any disease in a region a clear understanding of the disease’s background is essential [15]. Data collected through passive surveillance can provide information on the sources and nature of a disease in a region. In this manuscript, historical passive surveillance data sourced from Afghanistan are analyzed to determine the temporal and spatial distribution of FMD in Afghanistan.

## Results

### Summary

**Fig. 1:**
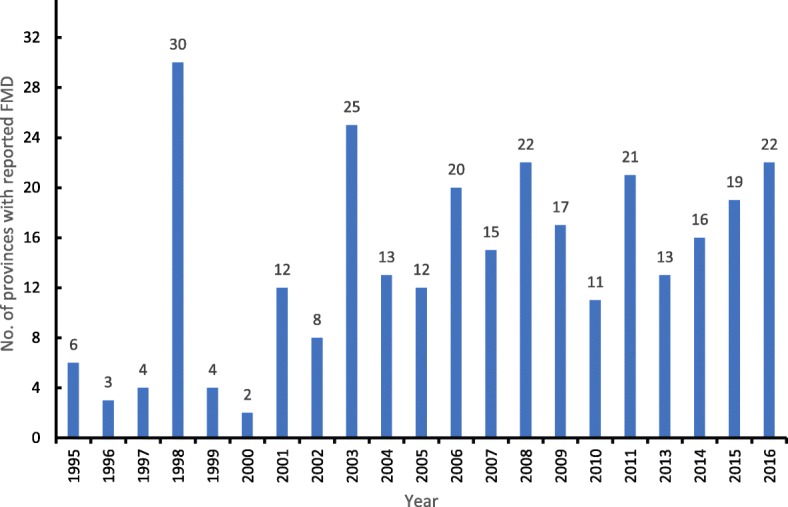
Number of provinces in Afghanistan with FMD cases/outbreaks for the years 1995–2011 and 2013–2016

A total of 4171 outbreaks of FMD were reported from 1995 to 2008 in Afghanistan, 4089 positive cases (54.1%; 95% CI, 53.0–55.2) were diagnosed from 7558 blood samples collected for the years 2009, 2011 and 2013 to 2015, and 574 clinically suspected cases were recorded in 2016. In 2010, 182 (83.49%; 95% CI, 77.9–88.2) of the 218 tissue samples collected were test positive. The disease was reported from an average of 14 provinces (range 2–30) each year during 1995 to 2011 and 2013 to 2016 (Fig. 1).

Data sourced for 2011 were the most complete of the studied years due to results being available from active surveillance undertaken in 21 provinces of the country. Additionally, in 2010 samples were collected for the identification of the circulating FMD virus serotypes within the country. Based on NSP 3ABC ELISA results, the distribution of FMD positive samples in the country was significantly different (χ^2^ = 263.98, df = 4, *P* < 0.001) between years (2009, 2011, 2013–2015) in which serological results were available. Of the 34 provinces present in Afghanistan, 30 (88.2%; 95% CI, 72.5–96.7) had reports documented of FMD during 1995–2008 with one or more cases of the disease from 2009 to 2016 (excluding 2012) being reported in 27 (79.4%; 95% CI, 62.1–91.3) provinces. The study results are summarized in Table 1.

**Table 1:** Summary of years, number of provinces, number of samples/outbreaks and tests used for diagnosis of FMD during the study period

Year	Number of samples /outbreaks	Number of provinces	Data type	Diagnostic process
1995	21	6	N/A	Clinical cases
1996	15	3	N/A	Clinical cases
1997	47	4	N/A	Clinical cases
1998	118	30	N/A	Clinical cases
1999	12	4	N/A	Clinical cases
2000	12	2	N/A	Clinical cases
2001	67	12	N/A	Clinical cases
2002	70	8	N/A	Clinical cases
2003	97	25	N/A	Clinical cases
2004	183	13	Blood	Indirect ELISA (NSP, 3ABC)
2005	50	12	Blood	Indirect ELISA (NSP, 3ABC)
2006	3322	20	Blood	Indirect ELISA (NSP, 3ABC)
2007	59	15	Blood	Indirect ELISA (NSP, 3ABC)
2008	98	22	Blood	Indirect ELISA (NSP, 3ABC)
2009	3166	17	Blood	Indirect ELISA (NSP, 3ABC)
2010	218	11	Tissue	RT-PCR
2011	3243	21	Blood	Indirect ELISA (NSP, 3ABC)
2013	121	13	Blood	Indirect ELISA (NSP, 3ABC)
2014	370	16	Blood	Indirect ELISA (NSP, 3ABC)
2015	658	19	Blood	Indirect ELISA (NSP, 3ABC)
2016	574	22	Field observation	Clinical cases

### Spatiotemporal distribution

#### 1995 to 2008

Between 1995 and 2008, when only outbreaks of the disease were documented, 4171 FMD outbreaks (annual mean = 298, median = 63) were reported (Table 2). During this period the highest number of reported outbreaks occurred in 2006 (*n* = 3322) followed by 2004 (*n* = 183) and 1998 (*n* = 118). Disease outbreaks were reported in the most provinces (88.2%) in 1998 followed by the years 2003 (73.5%), 2008 (64.7%) and 2006 (58.8%). There was a strong correlation between the number of provinces with outbreaks and the actual number of outbreaks reported (*r* = 0.85), and between the number of districts with outbreaks and the number of outbreaks reported (*r* = 0.68). There was no significant difference in the number of outbreaks reported over the study period (q = − 4.6, df = 2, *P* = 1). As these data were collected through passive surveillance based on clinically suspected cases with no exact time (month, season) and specific geographical location, no further analyses were possible to broaden the understanding of the disease’s epidemiology in the country during this period.

**Table 2:** Number of provinces and districts reporting FMD outbreaks in the 1995–2008 period

No	Year	No. of provinces that reported outbreaks of the 34 provinces present	No. of districts that reported outbreaks of the 402 districts present	Total No. of FMD outbreaks reported
1	1995	6	10	21
2	1996	3	5	15
3	1997	4	8	47
4	1998	30	114	118
5	1999	4	8	12
6	2000	2	6	12
7	2001	12	25	67
8	2002	8	26	70
9	2003	25	102	97
10	2004	13	39	183
11	2005	12	36	50
12	2006	20	171	3322
13	2007	15	29	59
14	2008	22	N.A	98
	Total			4171

#### 2009

**Fig. 2:**
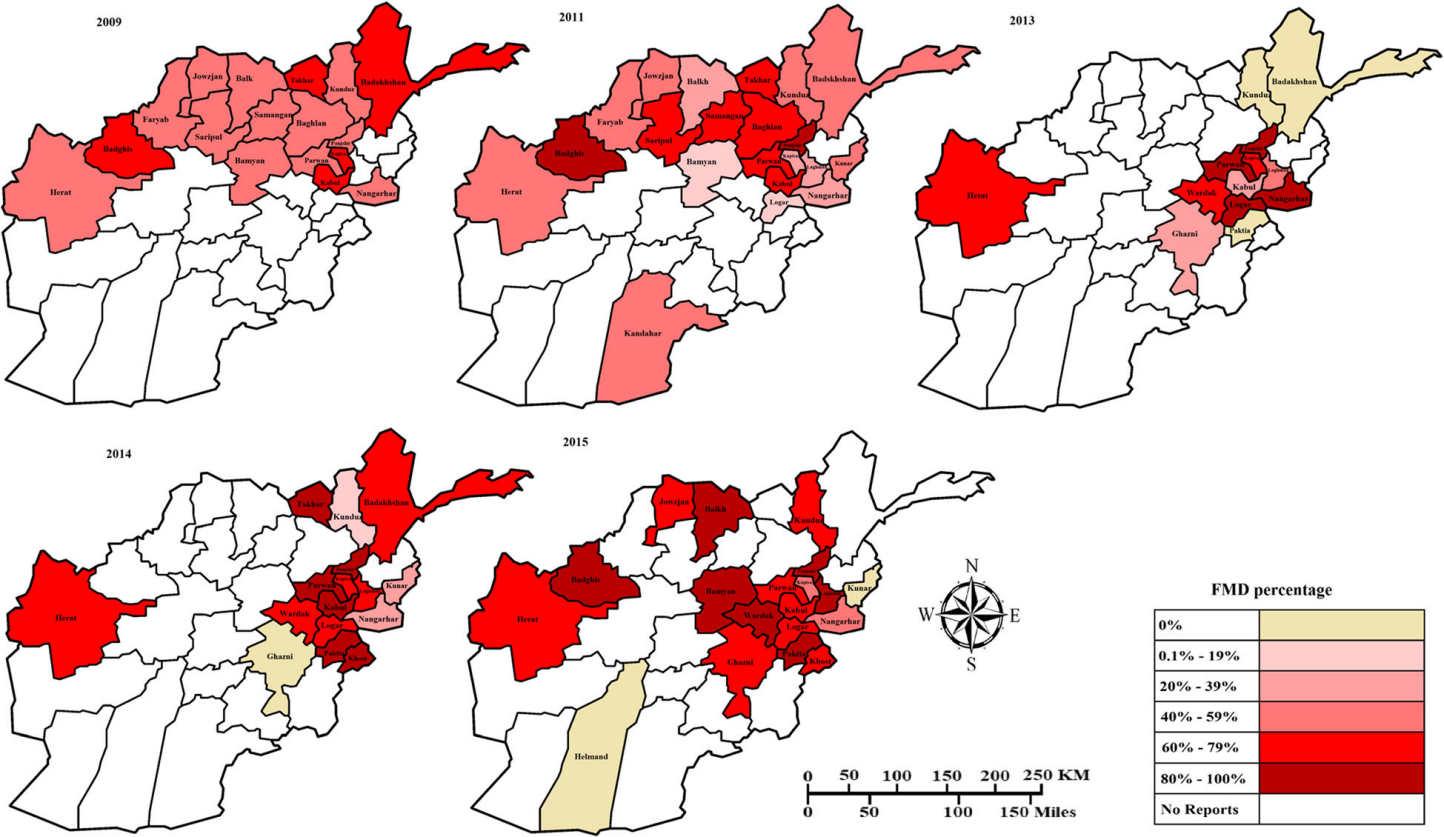
Choropleth maps of Afghanistan provinces showing the distribution of FMD based on serological results available for the years 2009, 2011, 2013–2015, generated by Arash Osmani (corresponding author)

A total of 3166 blood samples were collected from ruminants (cattle, sheep, goats and buffalos) from randomly selected villages by local VFU staff as part of their routine tasks in 17 of 34 provinces in Afghanistan in 2009. The distribution of FMD based on blood samples positive to FMD tested with a trapping-indirect ELISA for the detection of antibodies to non-structural protein (NSP) 3ABC of FMDV in the different zones sampled are displayed in Fig. 2.

**Fig. 3:**
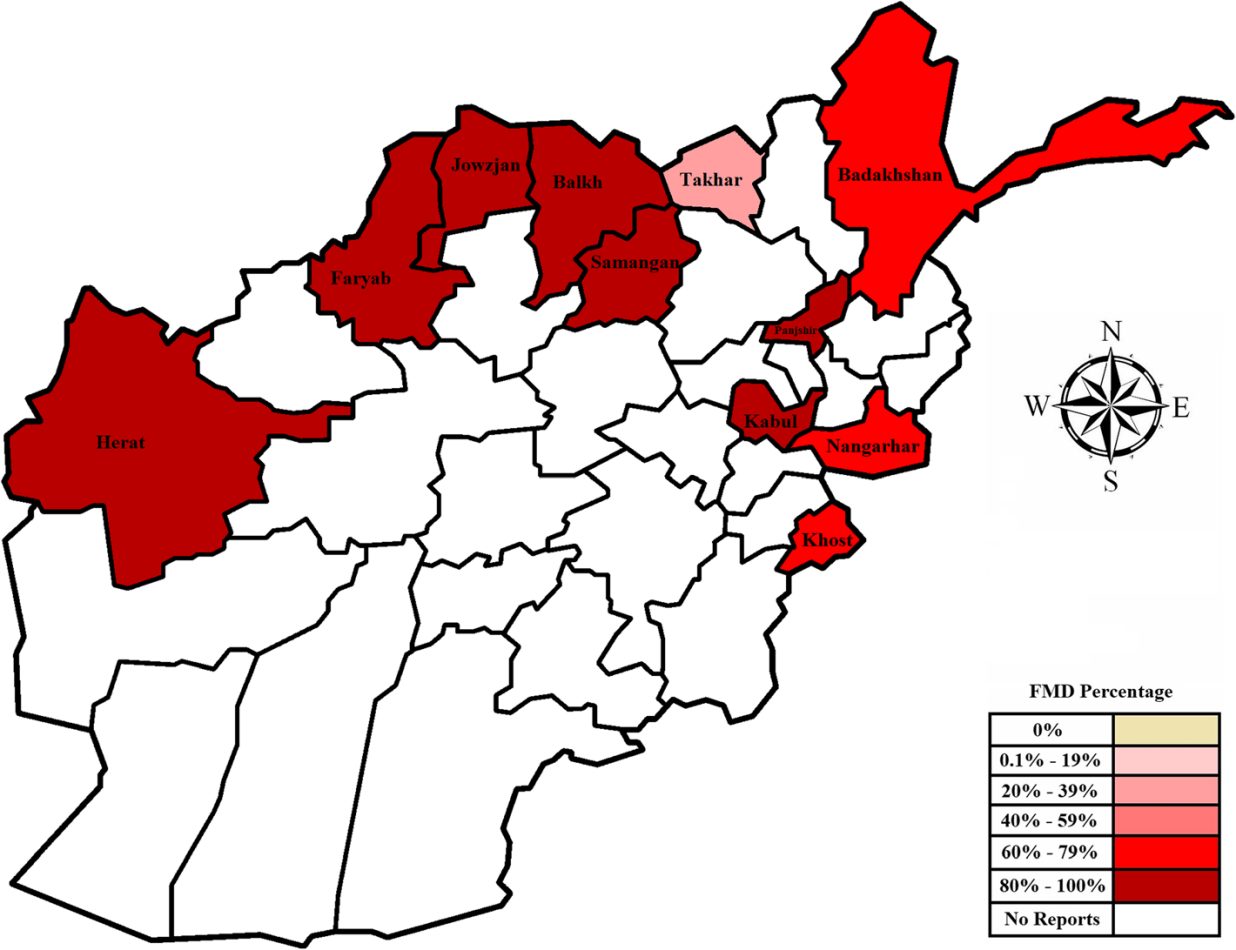
Provincial choropleth map of Afghanistan showing the distribution of FMD based on the percentage of tissue samples submitted in 2010, positive for FMDV genome on a RT-PCR, generated by Arash Osmani (corresponding author)

Overall 54.8% (95%; CI, 53.1–56.6) of samples were seropositive with significant differences between the zones (χ^2^ = 108.2, df = 4, *P* < 0.001) and the 17 provinces sampled (χ^2^ = 174.2, df = 16, *P* < 0.001). The highest seroprevalence (66.3%; 95% CI, 63.2–69.2) was found in the north-east zone and the lowest (41.9%; 95% CI, 38.2–45.7) in the east zone. Badakhshan province, in the north-east zone, had the highest seroprevalence (73.8%; 95% CI, 69.4–77.9) followed by Takhar also in the north-east (67.3%; 95% CI, 61.9–72.3). The highest seroprevalence in the central zone was recorded in Kapisa (66.7%; 95% CI, 59.5–73.3) and the highest in the west was Badghis (64.58%; 95% CI, 49.5–77.8). Panjshir province in the central and Nangarhar province in the east zones had the lowest seroprevalences (41.7%; 95% CI, 27.6–56.8; 41.9%; 95% CI, 38.1–45.7, respectively) (Fig. 3, Additional file 1).

#### 2010

In 2010, 218 samples of epithelial tissues were collected from cattle with clinical signs of the disease in 11 provinces. The samples were sent to the World Reference Laboratory (Pirbright, UK) for serotyping using FMDV genome detection by RT-PCR. Of these samples 83.5% (95% CI, 77.9–88.2) were positive. The percentages of positive tissue samples submitted from different provinces are displayed in Fig. 3. There was a significant difference in the proportion of test positive samples between zones (*P* < 0.001). Test positive samples were detected in all 11 provinces and the proportion positive was also significantly different between provinces (*P* = 0.026). All tissues sourced from the provinces of Samangan, Balkh, Jawzjan and Faryab in the north zone of the country were positive. In comparison, the lowest proportion of positive samples was from Takhar in the north-east zone (36.4%; 95% CI, 10.9–69.2) (Additional file 2).

#### 2011

A total of 3243 blood samples from ruminants (cattle, buffalo and sheep) were tested with a trapping-indirect ELISA for the detection of antibodies to NSP in 2011. Samples were submitted from 21 provinces situated in six of the eight zones of the country, with 46.4% testing positive (95% CI, 44.7–48.1). There was a significant difference between the number of test positive samples between zones (χ^2^ = 69.6, df = 5, *P* < 0.001) and also between provinces (χ^2^ = 353.6, df = 20, *P* < 0.001). The north zone had the highest percentage of positive samples (53.2%; 95% CI, 49.8–56.6) and the south-west zone the lowest (34.9%; 95% CI, 31.0–39.0). All 32 samples from Panjshir province in the central zone were test positive (100%; 95% CI, 89.1–100). Most of the samples from Badghis province in the west (86.42%; 95% CI, 80.2–91.3) and Samangan province in the north (76%; 95% CI, 66.3–84.2) zones of the country were also positive. Logar province in the south-west zone had the lowest seroprevalence (17.6%; 95% CI, 11.7–24.9), followed by Bamyan province in the central zone (18.4%; 95% CI, 11.6–26.9) (Fig. 2, Additional file 3).

#### 2013

In 2013 provincial VFU officers from 13 provinces collected 121 blood samples from ruminants (cattle, sheep, goats and buffalo) from randomly selected villages and submitted these to the CVDRL and CED. The percentages and distribution of samples seropositive to FMD tested with an NSP indirect ELISA in the different provinces are displayed in Fig. 2 and Additional file 4. Overall 67.8% (95% CI, 58.7–76.0) were seropositive. The seroprevalence was again significantly different between zones (χ^2^ = 17.5, df = 4, *P* < 0.001) and provinces (χ^2^ = 50.7, df = 12, *P* < 0.001). The highest seroprevalence was in the east zone (75%; 95% CI, 19.4–99.4) while the north-east zone had no seropositive results (0%; 95% CI, 0–41). All samples from Parwan in the central zone (100%; 95% CI, 2.5–100) and Nangarhar in the east zone (100%; 95% CI, 15.8–100), and most samples from Panjshir in the central (95%; 95% CI, 83.08–99.39) zone were seropositive. In contrast no samples from Badakhshan (0%; 95% CI, 0–97.5) and Kunduz (0%; 95% CI, 0–45.93) in the north-east zone and Paktia in the south zone (0%; 95% CI, 0–70.76) were test positive (Additional file 4).

#### 2014

A total of 370 blood samples from ruminants (cattle, sheep, goats and buffalo) were submitted to the CED and CVDRL of the Veterinary Directorate from 16 provinces in 2014 with 76.2% (95% CI, 71.5–80.5) positive (Additional file 5). The seroprevalence varied significantly between zones (χ^2^ = 21.6, df = 4, *P* < 0.001) with the central zone having the highest (84.4%; 95% CI, 77.5–89.8) and the east zone the lowest (43.5%; 95% CI, 23.2–65.5). Similarly, the seroprevalence varied between provinces (χ^2^ = 47.8, df = 15, *P* < 0.001) with all samples from Paktia (100%; 95% CI, 39.76–100) and Khost (100%; 95% CI, 15.81–100) in the south zone being positive. Kunar province in the east zone had the lowest seroprevalence (25%; 95% CI, 0.6–80.6) (Fig. 2, Additional file 5).

#### 2015

In 2015, 658 blood samples from ruminants (cattle, sheep, goats and buffalo) originating from 19 provinces were tested at the CVDRL with an ELISA for antibodies to NSP. The distribution and percentages of seropositive samples collected from different provinces are displayed in Fig. 2 and Additional file 6. Overall the seroprevalence was 73.9% (95% CI, 70.3–77.2) with significant differences between zones (χ^2^ = 14.64, df = 6, *P* = 0.02) and provinces (χ^2^ = 68.2, df = 18, *P* < 0.001). Out of the 7 zones from which blood samples were submitted, the north zone had the highest seroprevalence (89.7%; 95% CI, 75.8–97.1), while all samples from the south-west zone were negative (0%; 95% CI, 0–84.2). All samples from Maydan Wardak province in the central (100%; 95% CI, 85.8–100), Badghis in the north-west (100%; 95% CI, 39.8–100) and Paktia in the south (100%; 95% CI, 2.5–100) zones were seropositive. In contrast no samples from Kunar in the east (0%; 95% CI, 0–97.5) and Helmand (0%; 95% CI, 0–84.2) in the south-west zones were test-positive (Additional file 6).

#### 2016

**Fig. 4:**
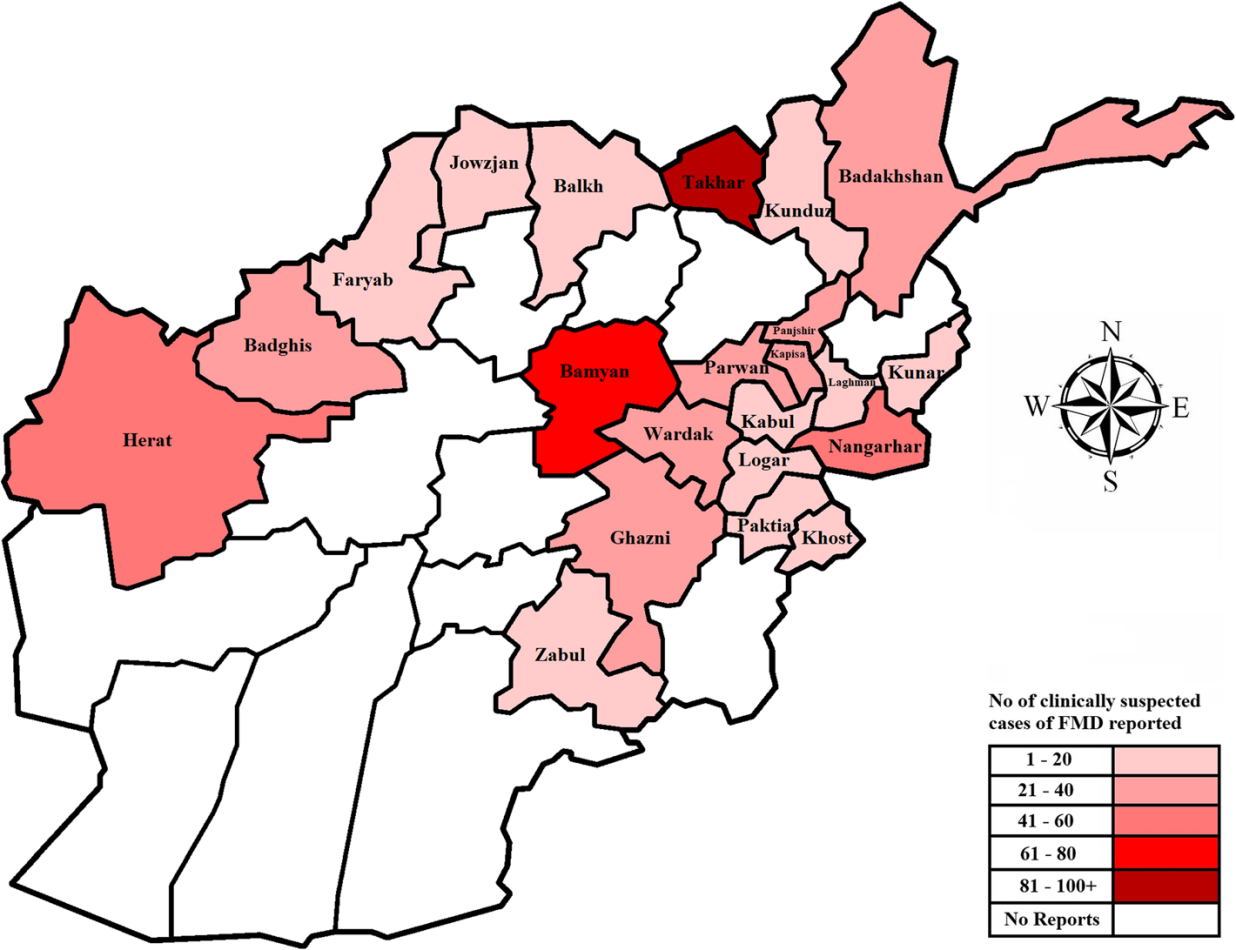
Provincial choropleth map of Afghanistan displaying the number of clinically suspected cases of FMD reported in 2016, generated by Arash Osmani (corresponding author)

In 2016 clinically suspect cases of FMD were reported to the CED of Veterinary Directorate MAIL. A total of 574 cases clinically suspected to be FMD were reported in 22 provinces (mean = 26, median = 21) and 115 districts consisting of 465 cases in cattle and 109 in sheep and goats. The number of clinically suspect cases of FMD reported in 2016 is displayed in Fig. 4. There was a substantial positive correlation (*r* = 0.70) between the number of districts with cases and the number of reported cases in 2016. The distribution of the reported cases was significantly different between zones (*P* = 0.019) and also between provinces (*P* < 0.001). More cases were reported from the central zone (36.9%; 95% CI, 33–41 of all cases) followed by the north-east zone (22%; 95% CI, 18.6–25.6), and the lowest number of cases was reported in the south-west zone (0.52%; 95% CI, 0.1–1.5) of all cases in the year (Additional file 7).

### Species affected

Cattle were involved in all of the outbreaks reported in Afghanistan during the last two and half decades.

In 2009 and 2011 data on the seroprevalence in the different livestock species were available and these data are summarized in Table 3. In each year, there was a significant difference between the percentage seropositive in the different livestock species (χ^2^ = 8.82, *P* = 0.002; and χ^2^ = 9.76, *P* = 0.007 for 2009 and 2011, respectively). Of the animals from which blood samples were collected the majority were cattle (92.4 and 96% of all samples in 2009 and 2011, respectively). Cattle also had the highest seroprevalence of the sampled species of 51.4% (95% CI, 49.6–53.1) in 2009 and 44.7% (95% CI, 43–46.4) in 2011.

**Table 3:** Comparison of seroprevalence using an NSP 3ABC indirect ELISA in different livestock species in 2009 and 2011

Year	Livestock type tested	Total number of samples tested	Percentage of seropositive samples (95% CI)	*P*-value for comparison between species
2009	Cattle	2926	51.4 (49.6–53.1)	0.002
	Buffalo	240	3.4 (2.8–4.1)	
		3166	54.8 (53–56.5)	
2011	Cattle	3112	44.7 (43–46.4)	0.007
	Buffalo	80	1.3 (0.9–1.7)	
	Sheep	51	0.4 (0.2–0.7)	
		3243	46.4 (44.6–48.1)	

### Serotypes of FMDV

Outbreaks of FMD from 1995 to 2016 in Afghanistan from which samples were collected were caused by serotypes A, O and Asia-1. The detection of different serotypes, as determined by the World Reference Laboratory for FMD (WRLFMD) in Pirbright, UK, for the period is summarized in Table 4.

**Table 4:** Historical reports on the FMDV types detected in Afghanistan which are available only on WRL database [7]

FMDV serotype	Years reported	
	Before 1996	1996–2017
O	1957, 1965, 1971–1972, 1974–1975	1996, 2004, 2007, 2009–2011, 2013–2014, 2016, 2017
A	1956, 1959, 1964, 1970, 1975	2004–2005, 2007, 2009–2011, 2013, 2016, 2017
C	1957	Not detected
Asia-1	1957, 1963, 1971–1972	2001, 2003–2004, 2009, 2011, 2013–2014, 2016, 2017
SAT1, SAT2, SAT3	Not detected	Not detected

### Consolidation of the results for the study period

The distribution of FMD in the country was significantly different (χ^2^ = 263.98, df = 4, *P* < 0.001) between the 2009, 2011 and 2013–2015 periods for which serological results were available. The seroprevalence was highest in 2014 and 2015 (76.2%; 95% CI, 71.5–80.5; 73.9%; 95% CI, 70.3–77.2, respectively).

The north zone had the highest seroprevalence in 2015 (89.7%; 95% CI, 75.8–97.1) followed by the central zone in 2014 (84.4%; 95% CI, 77.5–89.8). A comparison and summary of the results (distribution and percentages) for the 7/8 different zones in the different years of the study is summarized in Table 5 and Fig. 2. Of the 34 provinces in the country, Kabul, Hirat, Nangarhar and Panjshir had outbreaks of FMD reported for every year that laboratory data were available.

**Table 5:** Comparison of zones, years, percentages of FMDV test positive samples and clinically diagnosed cases of FMD between years 2009–2011 and 2013–2016 in Afghanistan

Years		2009	2010	2011	2013	2014	2015	2016
Sample type		Serum	Tissue	Serum	Serum	Serum	Serum	Clinical cases
Zones	Central	58.2%	95.6%	42.1%	76.2%	84.4%	74.3%	212
	North-East	66.3%	55.6%	47.9%	0.0%	75.9%	76.9%	126
	North	48.7%	100%	53.2%	–	–	89.7%	39
	North-West	–	–	–	70%	76.4%	71.8%	77
	West	53.4%	79.5%	52.5%	–	–	–	–
	South	–	–	–	58.8%	64.9%	72.4%	55
	South-West	–	–	34.9%	–	–	0.0%	3
	East	41.9%	64.7%	36.9%	75%	43.5%	60%	62
	Total	54.8%	83.5%	46.4%	67.8%	76.2%	73.9%	574

## Discussion

This is the first analysis of historical data on FMD in Afghanistan and was based on analyzing the results of outbreaks and blood samples collected from selected districts/provinces officially sampled (recorded) by the Afghan government during the period 1995 to 2016 [16]. From 2009 onwards samples of blood and tissue were submitted to the CED and CVDRL of the Veterinary Directorate through passive surveillance activities conducted by provincial VFU staff. Besides the ongoing conflict in Afghanistan, which has made it a challenge to undertake active disease surveillance, one of the issues with passive surveillance has been the potential biased nature of reporting, which is mostly dependent upon reporting of disease cases by farmers. Zafar [12] reported that infectious diseases, along with many other factors, including safety, socio-economic disruption, lack of a stable government and drought, especially the severe drought from 1998 to 2001 [17], affected livestock production and the surveillance of animal diseases in Afghanistan. The surveillance system implemented for most animal diseases in Afghanistan is of a passive nature [18]. The lack of disease surveillance networks [17, 19] to carry out disease investigations and the absence of an Epidemiology Unit to manage livestock information in such a way that can update and input into the development of the livestock industry, are considered major issues in Afghanistan [19]. As surveillance requirements and available resources vary enormously among countries [18], the surveillance system in Afghanistan needs to be developed within the current constraints present in the country.

The spatial analysis indicated that FMD was present in each year in the provinces of Nangarhar in the east zone, Kabul in the central zone and Hirat in the north-west zone. Hirat and Nangarhar share a porous border with Iran to the west and Pakistan to the southeast, respectively, and major trade routes traverse Kabul connecting this province in central Afghanistan to the rest of the country. Others [20, 21] have highlighted the spread of FMD through livestock movement and the disease has been reported to be endemic in Iran [22] and Pakistan [23, 24]. Hirat province is considered to be one of the main routes of FMD transmission to and from Iran when cattle and beef carcasses are imported from India and Pakistan to Afghanistan and through Afghanistan to Iran [25]. Consequently, there is a high probability of infection spreading between countries in the region through the movement of cattle, the continued movement of refugees across the border and significant festivals such as “Eid-ul-Adha” when many animals (mainly cattle) are imported legally and illegally into Afghanistan from Pakistan. The spread of FMD during Eid-ul-Adha festival is further supported by Anjum et al. [26] who reported that during June 2002 to June 2005 the majority of the FMD outbreaks in Pakistan (Afghanistan’s neighbor with the longest border) occurred around Eid-ul-Adha, which was due to the large number of animal movements. Even though Afghanistan legally imports large numbers of animals to meet the demand for meat, a large undetermined number are also smuggled across its borders [12]. This is further supported by the findings reported by Jamal et al. [1] who detected similar FMDV serotypes and topotypes in Afghanistan and Pakistan and proposed that this was linked with the uncontrolled and extensive movement of animals between the two countries. Similarly, Rweyemamu [25] highlighted the free flow of FMDV between Afghanistan and Pakistan. The seasonal movement of people with their animals to regions of different climate, described as transhumance by Macpherson [27], is one of the major contributors to the spread of FMD regionally and globally [28]. The east of Afghanistan (mainly Nangarhar province) is ecologically very similar to Pakistan and is linked to Pakistan by transhumance, trade and fattening enterprises [25]. Poor quarantine measures with inefficient quality control of animal products in Afghanistan also contributes to the spread and distribution of FMD. Ineffective quarantine measures and importation of animal products and live animals, especially from Pakistan, play a role in the regional spread of transboundary animal diseases, such as FMD [29] Therefore, to understand the spread of FMD in Afghanistan it is necessary to determine the movement, both legal and illegal, of animals within the country and with neighboring countries.

The temporal analysis of the available historical data indicated that FMD is endemic in Afghanistan, with outbreaks occurring in every year data were available. However, the level of disease/infection detected is likely to be an underestimate of the real situation due to underreporting of the disease. The potential for underreporting is not surprising given the unstable socio-political situation during the study period, especially during and after the Taliban regime in the country, when the animal husbandry sector, as with other sectors of the country, was largely disrupted. The long lasting lack of stability in the country has significantly contributed to the worsening animal health status in Afghanistan [19]. Although there was no official research conducted on FMD in Afghanistan during this period, Jamal et al. [13] reported a prevalence of 89.4% (95% CI, 76.9–96.5) in Afghanistan as a result of findings of testing oral swabs and epithelial samples from 157 suspected cases between 2002 and 2009 in Afghanistan and Pakistan. Additionally, Jamal et al. [24] conducted research on the genetic characterization of FMDV from clinically healthy cattle and buffalo in endemic locations (live animal markets in Kabul, Badakhshan and Nangarhar) between July 2008 and August 2009, reporting a prevalence of 12.2% (95% CI, 7.8–17.9). This and the current study supports the endemic nature of the disease in the country with porous borders, animal markets, and poor maintenance of vaccines all contributing to the regular occurrence of FMD in Afghanistan [17]. Since Afghanistan imports live animals, especially cattle and buffalo, for immediate slaughter or slaughter within a week of arrival from Pakistan [30], it is likely that the circulating strains of FMDV in Afghanistan originate from and are similar to those in Pakistan.

Cattle were the main species affected with FMD in Afghanistan during this study, being involved in all outbreaks investigated. Cattle are considered the most susceptible ruminant, in comparison to sheep and goats which are relatively resistant to the virus [31, 32]. However the susceptibility of sheep and goats does vary between different breeds and also with different virus strains [32]. Sheep are often involved in disseminating FMDV, both between and within countries, with FMDV persisting in these animals for up to 12 months, while in goats FMDV is estimated to persist for 2–3 months [33]. However, Aftosa [31] questioned the ability of small ruminants to maintain FMDV in the absence of cattle. Furthermore, FMDV has been reported to persist for as long as 3.5 years [34] in clinical and subclinical cases in cattle [35]. Thornley, France [36] reported that, for a given exposure to virus, cattle are approximately 15 times more likely to become infected than sheep, and an infected cow will shed twice as many virus particles than an infected sheep, highlighting the importance of this species in FMD transmission.

From the limited data available, FMDV serotype O was found to be the leading serotype in the country as has also been reported by others [1, 5, 6, 25].

Although the data in this paper represents an important contribution to the recognition of the history and epidemiology of FMD in Afghanistan, it must be acknowledged that: it does not present all activities conducted on FMD during the study period and only includes activities that were officially recorded in government documents and has minimal data sourced from research; the quality of the data used in this nationwide review varies in type, quality and quantity between years; minimal data were available for the period 1995 to 2008 (Table 2); there is likely to be bias in the reporting of disease in government records (for instance: the presence of a huge difference in the number of outbreaks between 2006 and the other years during the period 1995–2008); and may not represent all Government data due to a reluctance of the government authorities to share all of their data.

## Conclusion

The study reported in this paper has evaluated the spatiotemporal distribution of FMD based on data sourced from the Afghan government official records, along with other publicly available records. Gathering nation-wide data about FMD from a single source in Afghanistan is challenging, particularly due to the absence of a well-functioning official disease database in the country [18]. Based on the few reports available at CED, which all originated through passive surveillance activities, it is extremely likely that FMD is underreported within the country and there is a need to improve the surveillance system in the country. However, passive data collected over the last two and half decades have provided some knowledge and insight into FMD in Afghanistan [16]. This allows national and international organizations to focus on the disease through assisting and improving the country’s ability to control the disease. Nevertheless, improved knowledge about the disease, quality field activities and strong governmental support to control the disease is not enough on its own and there is the need for expanded administrative activities and further research, including undertaking field studies, to effectively control the disease. The current analysis focused on the spatiotemporal distribution of FMD in Afghanistan and highlighted the need for further statistical and empirical analyses to be conducted to ensure suitable control procedures are developed, implemented and subsequently evaluated. To control the disease, it is important to identify risk factors associated with the disease. Additionally, information about animal movement patterns, governmental approach to animal transboundary disease and quarantine and control measures are key factors to be addressed for a suitable control program of FMD in Afghanistan.

## Methods

### Source of data

#### 1995–2008

The data presented in this paper for the years 1995 to 2008 were gathered from unpublished government and non-government documents. Reports on FMD during these years were limited and only recorded as outbreaks and number of affected districts and provinces (Table 2).

#### 2009–2011

Data for the years 2009 to 2011 were taken from the final reports of the FAO Regional Project of Controlling Transboundary Animal Diseases (TADs) in Central Asian Countries (GTFS/INT/907/ITA) FAO, Afghanistan. Data from the TADs project were obtained from the Central Veterinary Diagnostic and Research Laboratory (CVDRL). The surveys were coordinated jointly by the FAO and the Central Epidemiology Department (CED) within the Directorate of Veterinary Services at Ministry of Agriculture, Irrigation and Livestock (MAIL). Data for 2010 relates to the results from testing oral swabs and epithelial samples collected from clinically suspected cases, and those of 2009 and 2011 were from serological samples collected during sero-surveillance (Table 1).

#### 2012

Based on personal communications with the Head of the CED, there was no activity relating to FMD in 2012.

#### 2013–2016

Reports of FMD for 2013 to 2016 were provided by the CVDRL and CED of the Veterinary Directorate of MAIL. From 2013 to 2015, the provincial Veterinary Field Units (VFU) and veterinary professionals diagnosed cases of FMD through the collection and submission of samples to the CVDRL. In 2016 the number of cases recorded was based on the presence of clinical signs reported by local VFU staff (Table 1).

#### Serotypes

Precise serotype specific data for the study period (1957–2016) along with data from 2017 were sourced from the World Reference Laboratory for Foot-and-Mouth Disease (WRLFMD) website (http://www.wrlfmd.org/western-and-central-asia/afghanistan).

A case was defined as an animal displaying clinical signs or lesions characteristic of FMD with or without confirmatory laboratory diagnosis. An outbreak was defined as the occurrence of one or more cases of FMD in a herd, village, district or province within a defined time period (month/season) that was reported to the authorities. Since FMD is endemic throughout Afghanistan, the use of the term case is preferred to the term outbreak with respect to the FMD status in the country, and currently the term “case” is used by the veterinary professional throughout the country. However, data from 1995 to 2008 are recorded as outbreaks, as this was the term used in the reports submitted by the provincial VFU staff during that time.

Due to a lack of information about the exact location names (village names, district names) of affected herds in most years (1995–2008), data were reported only as number of outbreaks and number of districts/provinces with outbreaks (Table 2).

**Fig. 5:**
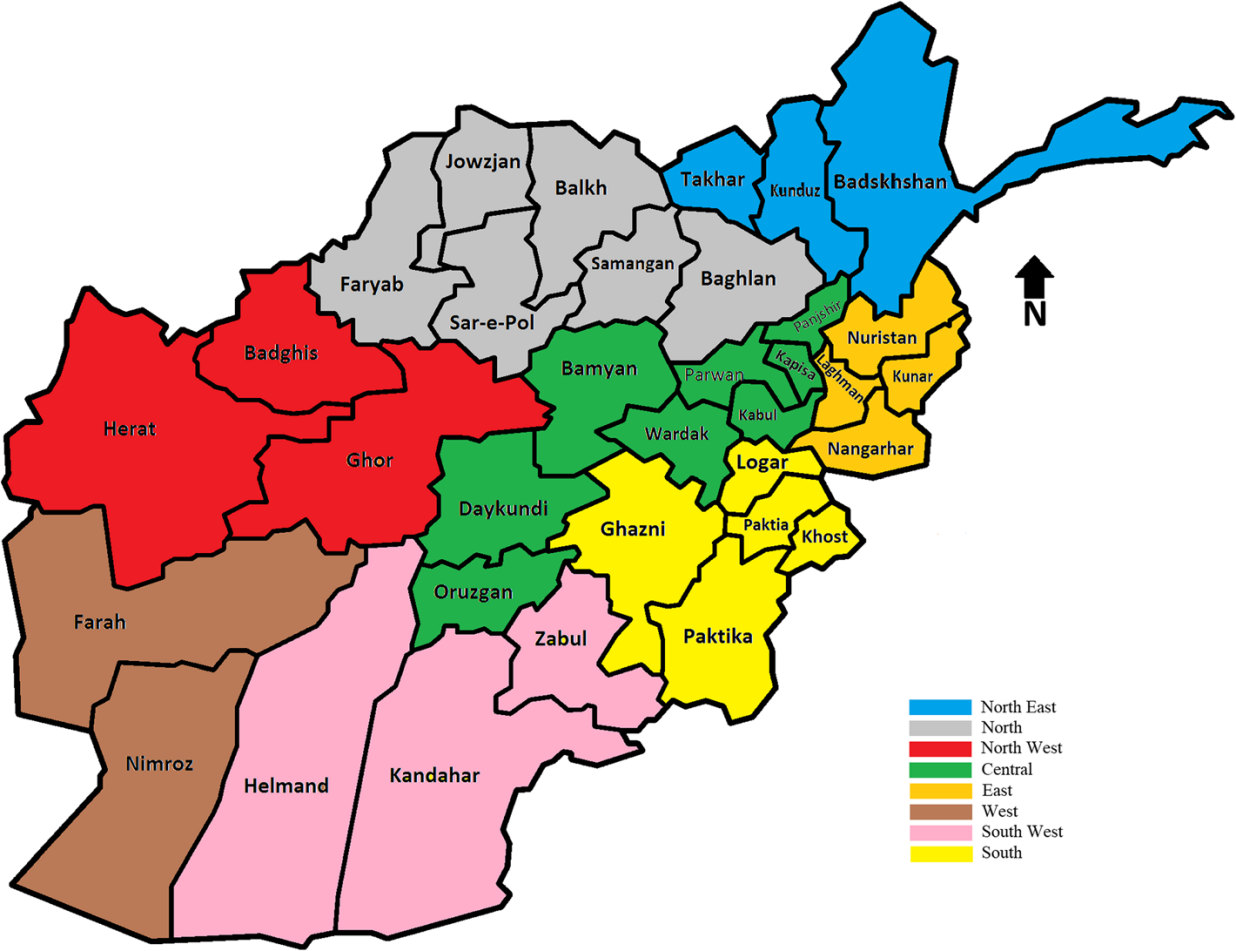
Map of Afghanistan displaying provinces and zones for animal disease surveillance, generated by Arash Osmani (corresponding author)

In this paper a Veterinary Professional in Afghanistan is defined as one of the following: 1) Veterinarians with a Doctor of Veterinary Medicine (DVM) degree or above; 2) A veterinary assistant with a 2-year diploma in animal science; 3) A para-professional (para-vet) with a 6 to 12 month certificate in animal science and veterinary medicine; and 4) A Basic Veterinary Worker (BVW) who had undergone one to 4 weeks of basic clinical handling of animals. For the purpose of disease surveillance programs in 2009, Government and international agencies working on infectious diseases have divided the 34 provinces of the country into seven zones which in 2011 were expanded to eight zones (North-East, North, North-West, Central, East, West, South and South-West - Fig. 5).

### Statistical and computational analysis

All data were analyzed in Microsoft Excel (Microsoft® Excel for Mac, Version 15.29.1 “161,215”,© 2016 Microsoft), and RStudio (Version 1.1.383 –© 2009–2017 RStudio, Inc.) for Mac. Maps showing the spatial distribution of outbreaks were generated using Picture It (Microsoft Picture It! Library 10 “V10.0.612.0”© 1997–2004 Microsoft Corp.) and Microsoft Windows 2010 Paint (Version 1607 “OS Build 14,393.953© 2016 Microsoft Corporation).

#### 1995–2008

Limited data on the number of FMD outbreaks and number of districts and provinces reporting outbreaks were available in this period. Cochran q-test was used to determine the statistical significance of differences in the number of reported outbreaks in districts and provinces between years. The Spearman rank correlation coefficient test was used to evaluate the correlation between the number of districts/provinces with outbreaks and the number of outbreaks in different years.

#### 2009–2016

Descriptive statistics for laboratory data (2009–2011 and 2013–2015) and field reports (2016) were calculated. Estimates of the proportion of positive results of all samples and their 95% confidence intervals (95%CI) were calculated using a web based statistical calculator (www.vassarstats.net) and the confidence intervals for binomial proportions and Poisson rate estimation estimated using the method of Ross [37]. For data distribution analyses, the Kruskal-Wallis ANOVA, Chi-square and Cochran q-tests were used to determine statistical differences in the incidence of outbreaks in different zones/provinces between years. Odds ratios (OR) and their 95%CI were also calculated to measure the magnitude of association between test positive samples and provinces/zones. The Spearman rank correlation coefficient test was used to evaluate the correlation between the number of districts/provinces with outbreaks and the number of outbreaks in different years.

### Diagnostic tests

A trapping-indirect ELISA was used to detect antibodies to the non-structural (NS) polypeptide 3ABC of FMDV in the serum or plasma samples from large and small ruminants. The test’s specificity exceeds 99.5% with the sensitivity varying dependent upon the time lapsed after infection and on the previous vaccination status. Sensitivity as high as 100% for non-vaccinated cattle exposed to infection and 86.4% for the detection of carriers in vaccinated cattle have been reported [38]. Importantly the detection of anti-3ABC antibodies is indicative of a present or past infection with any of the seven FMDV serotypes, and vaccination does not induce a positive response to this test.

## Supplementary information

**Additional file 1.** Effect of zone on seroprevalence of FMD based on samples collected in 2009 and tested with an NSP indirect ELISA.

**Additional file 2.** Influence of zone and province on percentage of FMD positive tissue samples collected from cattle with clinical disease in 2010.

**Additional file 3.** Seroprevalence to FMD in different zones and provinces based on surveillance undertaken in 2011.

**Additional file 4.** Seroprevalence based on an NSP indirect ELISA for samples submitted in 2013 from different provinces in Afghanistan.

**Additional file 5.** Seroprevalence of FMD based on samples tested with an NSP indirect ELISA submitted in 2014from different provinces of Afghanistan.

**Additional file 6.** Seroprevalence based on an NSP indirect ELISA for samples collected in 2015 from different provinces in Afghanistan.

**Additional file 7.** Distribution of clinically diagnosed cases of FMD in cattle, sheep and goats in different provinces of Afghanistan in 2016.

## Data Availability

The corresponding author (AO), as an employee of the Veterinary Directorate, Afghanistan, had access to data on animal diseases from his country from the listed sources, and as part of his duties was required to analyse these data. Public access to these data from non-employees is only possible through written permission of the Veterinary Directorate of MAIL. However, all data generated or analysed during this study are included in this paper and in additional files attached to this paper. Additionally, this paper is part of a chapter in a thesis to be submitted for the fulfilment of the requirements for the award of Doctor of Philosophy of Murdoch University. The thesis will be available for public access on the online repository of Murdoch University after final examination (https://researchrepository.murdoch.edu.au/).

## Abbreviations

CED: Central Epidemiology Department
CVDRL: Central Veterinary Diagnostic and Research Laboratory
ELISA: Enzyme-Linked Immunosorbent Assay
FMD: Foot and Mouth Disease
FMDV: Foot and Mouth Disease Virus
MAIL: Ministry of Agriculture, Irrigation and Livestock
NSP: Non-Structural Protein
RT-PCR: Reverse Transcription Polymerase Chain Reaction
VFU: Veterinary Field Unit
WRLFMD: World Reference Laboratory for Foot-and-Mouth Disease
